# A deep analysis using panel-based next-generation sequencing in an Ecuadorian pediatric patient with anaplastic astrocytoma: a case report

**DOI:** 10.1186/s13256-020-02451-4

**Published:** 2020-08-31

**Authors:** Jennyfer M. García-Cárdenas, Ana Karina Zambrano, Patricia Guevara-Ramírez, Santiago Guerrero, Gabriel Runruil, Andrés López-Cortés, Jorge P. Torres-Yaguana, Isaac Armendáriz-Castillo, Andy Pérez-Villa, Verónica Yumiceba, Paola E. Leone, César Paz-y-Miño

**Affiliations:** 1 Centro de Investigación Genética y Genómica, Facultad de Ciencias de la Salud Eugenio Espejo, Universidad UTE, Quito, Ecuador; 2 Departamento de Cirugía Oncológica, Hospital Oncológico Solón Espinosa Ayala, Quito, Ecuador

**Keywords:** Pediatric anaplastic astrocytoma, High-grade gliomas, TP53, Li-Fraumeni syndrome

## Abstract

**Background:**

Anaplastic astrocytoma is a rare disorder in children from 10 to 14 years of age, with an estimated 0.38 new cases per 100,000 people per year worldwide. Panel-based next-generation sequencing opens new possibilities for diagnosis and therapy of rare diseases such as this one. Because it has never been genetically studied in the Ecuadorian population, we chose to genetically characterize an Ecuadorian pediatric patient with anaplastic astrocytoma for the first time. Doing so allows us to provide new insights into anaplastic astrocytoma diagnosis and treatment.

**Case presentation:**

Our patient was a 13-year-old Mestizo girl with an extensive family history of cancer who was diagnosed with anaplastic astrocytoma. According to ClinVar, SIFT, and PolyPhen, the patient harbored 354 genomic alterations in 100 genes. These variants were mostly implicated in deoxyribonucleic acid (DNA) repair. The top five most altered genes were *FANCD2*, *NF1*, *FANCA*, *FANCI*, and *WRN.* Even though *TP53* presented only five mutations, the rs11540652 single-nucleotide polymorphism classified as pathogenic was found in the patient and her relatives; interestingly, several reports have related it to Li-Fraumeni syndrome. Furthermore, *in silico* analysis using the Open Targets Platform revealed two clinical trials for pediatric anaplastic astrocytoma (studying cabozantinib, ribociclib, and everolimus) and 118 drugs that target the patient’s variants, but the studies were not designed specifically to treat pediatric anaplastic astrocytoma.

**Conclusions:**

Next-generation sequencing allows genomic characterization of rare diseases; for instance, this study unraveled a pathogenic single-nucleotide polymorphism related to Li-Fraumeni syndrome and identified possible new drugs that specifically target the patient’s variants. Molecular tools should be implemented in routine clinical practice for early detection and effective preemptive intervention delivery and treatment.

## Background

Brain tumors are the most common solid tumors in children, with gliomas being the largest component of these cancers [1, 2]. High-grade pediatric malignant gliomas are primarily anaplastic astrocytomas (AAs; World Health Organization [WHO] grade III) and glioblastomas (WHO grade IV) [2–6]. Pediatric AA is a rare disorder in children from 10 to 14 years of age, with an estimated 0.38 new cases per 100,000 people per year worldwide [7]. The natural history, causative genetic mutations, and brain locations of high-grade gliomas in the pediatric population differ from adult gliomagenesis [1, 4]. Currently, the only established risk factors are neurofibromatosis types 1 and 2, Turcot syndrome, and Li-Fraumeni syndrome (LFS) [3, 8]. The overall survival (OS) is low, with 5-year survival rates of less than 20% in affected individuals; where the extent of surgical resection is a prognostic factor, the survival rate decreases when tumors are inaccessible for surgical interventions [2, 3].

Environmental etiologies associated with pediatric high-grade gliomas are not clearly established [3, 4]. The signs and symptoms vary, depending on the location and aggressiveness of the tumor and on the age of onset [3]. Symptoms are nonspecific; they could be the result of intracranial pressure such as headache, nausea, and vomiting [9]. However, clinical manifestations could include unilateral paresis, monoparesis, hemisensory loss, dysphasia, aphasia, irritability, a change in feeding pattern, and impairment of recent memory [3, 9]. Symptoms present early in high-grade gliomas, causing focal neurologic deficits due to infiltration of normal tissue [3].

Surgical resection is the main management procedure for high-grade gliomas; it allows the acquisition of a tumor sample for pathological diagnosis, relieves intracranial pressure, and reduces tumor size [3]. Although complete resection is consistently associated with better prognosis [3, 10], it is challenging to achieve. Challenges arise due to the facts that brain tumor boundaries are hard to identify and surgery could compromise neural function [3, 4].

Traditional treatment consists of adjuvant chemotherapy and radiation for children older than 3 years of age; however, there are several studies that have incorporated multidrug therapies. Due to a lack of clear chemotherapy standards, the benefits of this treatment are still controversial [3, 11, 12]. Also, there are several long-term consequences that are not directly the effect of the tumor but are associated with radiotherapy, such as late cognitive and neuropsychological sequelae, endocrine abnormalities, and vasculopathies. Additionally, these patients require long-term follow-up due to the high risk of secondary malignancies [3].

These poor outcomes and long-term sequelae have led to the use of high-throughput technologies, which have yielded tremendous understanding of molecular tumorigenesis, better prognosis markers, predictors of response, and personalized treatment [6, 13, 14]. For instance, genomic studies reported that *PI3K* mutations can activate the (*RTK*)-*PI3K-MAPK* pathway involved in several human diseases, including cancer, neurological diseases, diabetes, and others [6]. Wu *et al.* also discovered alterations in *TP53* and *Rb*, which are related to cell cycle regulation [15]. Finally, in 2016, the WHO added molecular parameters to classify tumors of the central nervous system [16]. High-grade glioma classification is based on epigenetic patterns, copy number variations, and genetic mutations (somatic alterations) [17, 18]. *H3.3* (*H3F3A*) mutation at G34 usually is present in patients between the ages of 14 and 18 years old with a tumor in the cerebral cortex. Also, G34 mutation usually co-occurs with alterations in *TP53*, *ATRX*, and high levels of hypomethylation across the whole genome. Besides, the H3.3G34 subtype has a poor response to treatment, but prognosis is not as poor as that of patients with a mutation in histone 3.1 or 3.3 K27M. On the other hand, *IDH1* mutation is almost exclusively found in older adolescents (median age 16 years). Often, patients with *IDH1* mutations also present with alteration in genes such as *TP53*, *MGMT* promoter methylation, and G-CIMP (cytosine-phosphate-guanine [CpG] island methylator phenotype). The OS varies in patients with wild-type *IDH1* from those mutated; that is, the wild-type genotype has a 20% lower OS rate [18]. Additionally, recent studies also suggest using genome-wide deoxyribonucleic acid (DNA) methylation arrays and targeted sequencing. These two techniques can help clarify the diagnosis of pediatric AA [13].

Molecular profiling is revolutionizing cancer diagnostics and leading to personalized therapies. Several clinical trials are targeting new pathways [13]. For instance, *Cyclin D1* inhibition has been attempted with pembrolizumab, a humanized monoclonal antibody designed to block the action of the receptor PD-1 [19]. Another example is veliparib, a drug designed to block ribose polymerases [poly(ADP-ribose) polymerases], which are proteins involved in repairing DNA mutations. If a tumor cell is not able to repair itself, it may stop growing. Only patients without *H3*, *K27M*, or *BRAFV600* mutations were recruited to participate in one study [20]. However, clinical trials in children are rare. Therefore, in several diseases, the treatment for children is an extrapolation of clinical trials in adults. AA is not an exception, even though numerous studies have reported unpredictable and tragic effects in children due to their age-related biological characteristics.

The present study is important for two main reasons: (1) next-generation sequencing (NGS) technology in underdeveloped countries is scarce, given the high cost, which makes it currently almost impossible to apply clinically, and thus, patients remain with a presumptive diagnosis, receive inaccurate treatments, and have an undetermined prognosis; and (2) the aforementioned scenario is exacerbated by the lack of ethnically diverse oncological research [21]. Guerrero *et al.* showed that Latin American individuals are underrepresented in most basic oncological research as well as in cell lines, biobanks, cancer genomics, and clinical trials [22]. Therefore, to the best of our knowledge, our present study is the first in which a panel-based NGS of a mestizo AA child was used, highlighting its importance in increasing knowledge of basic cancer research.

## Case presentation

**Fig. 1:**
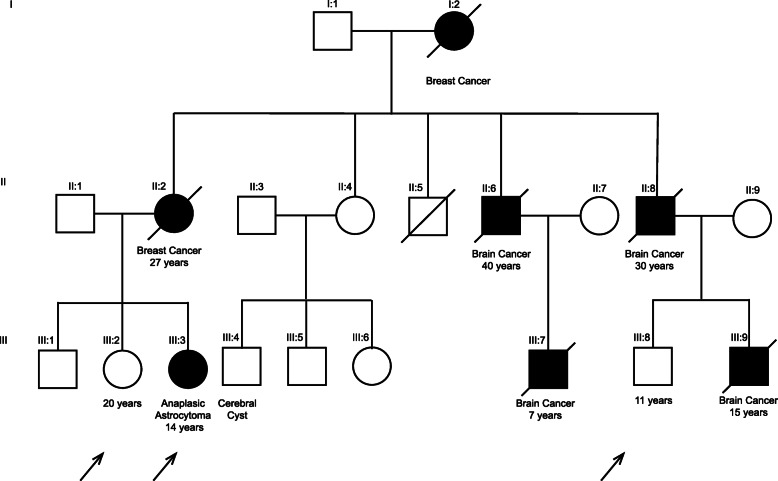
Genealogical tree of the family with Li-Fraumeni syndrome phenotype. Probands are indicated by the arrows (III:2, III:3, and III:8). Next-generation sequencing analysis found rs11540652 on *TP53* in the three probands. Subsequent genetic testing was offered to individuals III:1 and II:4

Our patient was a Mestizo girl with 63.1% Native American, 30.3% European, and 6.6% African ancestry, according to the latest report of the Ecuadorian population ethnicity [21]. She was a 13-year-old with multiple family background of cancer. Her mother had had breast cancer (right breast; estrogen receptor–positive/progesterone receptor–positive/human epidermal growth factor receptor–negative) during pregnancy; thus, our patient had been born prematurely. In addition to this, her maternal grandmother was also diagnosed with breast cancer, and two maternal uncles and two maternal cousins had central nervous system cancer types: glioblastoma and medulloblastoma (Fig. 1). The patient, with normal phenotypic features, presented with bilateral hearing loss from the age of 7 requiring hearing aids and language therapies. No family member has a medical history of deafness.

In January 2017, at 13 years old, she presented with headaches, nausea, photophobia, and phonophobia and was not only diagnosed with juvenile migraine but also treated with 400 mg of oral ibuprofen by her pediatrician. One year later, in November 2018, the patient went to the emergency room with a localized and intense pain in the frontal region, irradiation to the eyeballs, with a visual analog scale score of 9, accompanied by projectile vomiting. Her blood pressure was 130/80 mmHg; her heart rate was 80 beats per minute; and her body temperature was 36.4 °C. During her neurological examination, she was conscious and oriented with poor language. She obeyed simple and complex orders and had an isovolumetric process of 3 mm, light-reactive pupils, full-range eye movements, normal primary gaze, unchanged cranial nerves, normal mobility of upper and lower extremities, normal flexor plantar reflex, conserved superficial sensation, and no signs of meningeal irritation. In addition, laboratory test results showed no alterations in her complete blood count in both white and red cells as well as in platelets. However, a contrast-enhanced computed tomography of her brain revealed a right frontal hypodense mass of 4.5 × 3 × 1.5 cm, perilesional edema, compression of the frontal horn of the right lateral ventricle, and brain midline shift. The mass was located in the first right frontal gyrus, producing endocranial hypertension; this required an emergency right frontobasal craniotomy with tumor excision.

The patient’s postoperative evolution was satisfactory. She was treated with oral paracetamol 500 mg and oral oxycodone 10 mg, both administrated every 8 hours. Forty-eight hours after surgery, contrast-enhanced magnetic resonance imaging (MRI) of the brain was performed, which showed a 1-cm tumor remnant. She was discharged on the fifth postoperative day without neurological sequelae. Two weeks after surgery, her Karnosfky index dropped to 90% because she presented with sporadic holocranial headache that improved with pain relievers. Her physical examination did not reveal any sequelae in her gait or in her superior mental functions.

Immunohistological staining revealed the presence of astroprotein (glial fibrillary acidic protein) in the tumor cells. Furthermore, histopathological findings revealed Ki-67 positivity in 50% of tumor cells. The pathological diagnosis was AA (WHO grade III). On the other hand, genetic studies for mutations in *IDH1* and/or other genes were not conducted, owing to the fact that the hospital does not perform these types of assays. However, considering the age of the patient and her family history, doctors thought over the possibility that the origin of this tumor corresponded to a germline mutation. Therefore, she was referred to our research center with two other asymptomatic members of the patient’s family for NGS analysis (Fig. 1).

**Fig. 2:**
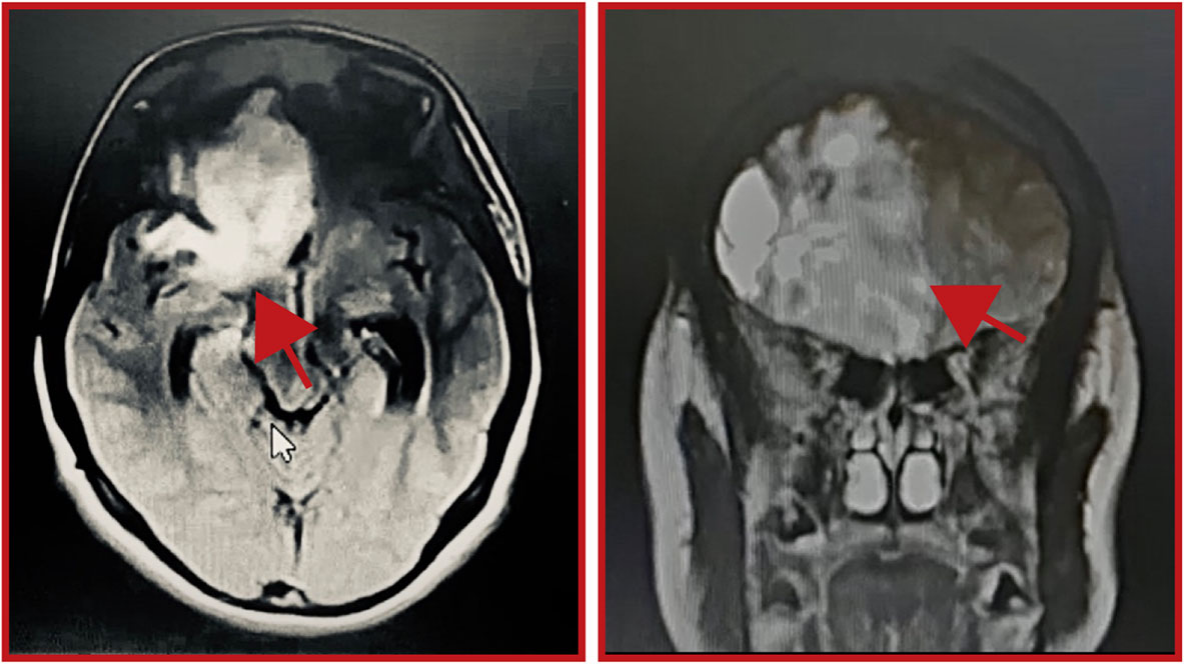
Contrast-enhanced tomography showing recurrent frontobasal tumor. *Red arrows* indicate the location of the tumor in axial axis (**a**) and coronal axis (**b**). Mouse cursor in (**a**) is irrelevant

The 1-cm tumor remnant is considered a poor prognosis factor. In January 2019, she started adjuvant therapy, which consisted of intensity-modulated radiation therapy with a total dose of 60 Gy in fractions of 2.0 Gy. On March 25, chemotherapy with oral Temozolomide was started at a dose of 200 mg/m^2^/day for 5 days, every 28 days, for 10 cycles. During this period, no treatment toxicities were recorded. However, on January 21, 2020 (that is, 4 weeks after receiving cycle 10 of Temozolomide), the patient was admitted to the emergency room. She presented with severe headache and vomiting, normal vital signs, without neurological deterioration or signs of endocranial hypertension. She was treated with opioids, and contrast-enhanced MRI of the brain showed an increase of the tumor size, which extended to the corpus callosum and left entorhinal cortex. The tumor was 8.7 cm in diameter with areas of necrosis and deviation from the midline to the left (Fig. 2).

**Fig. 3:**
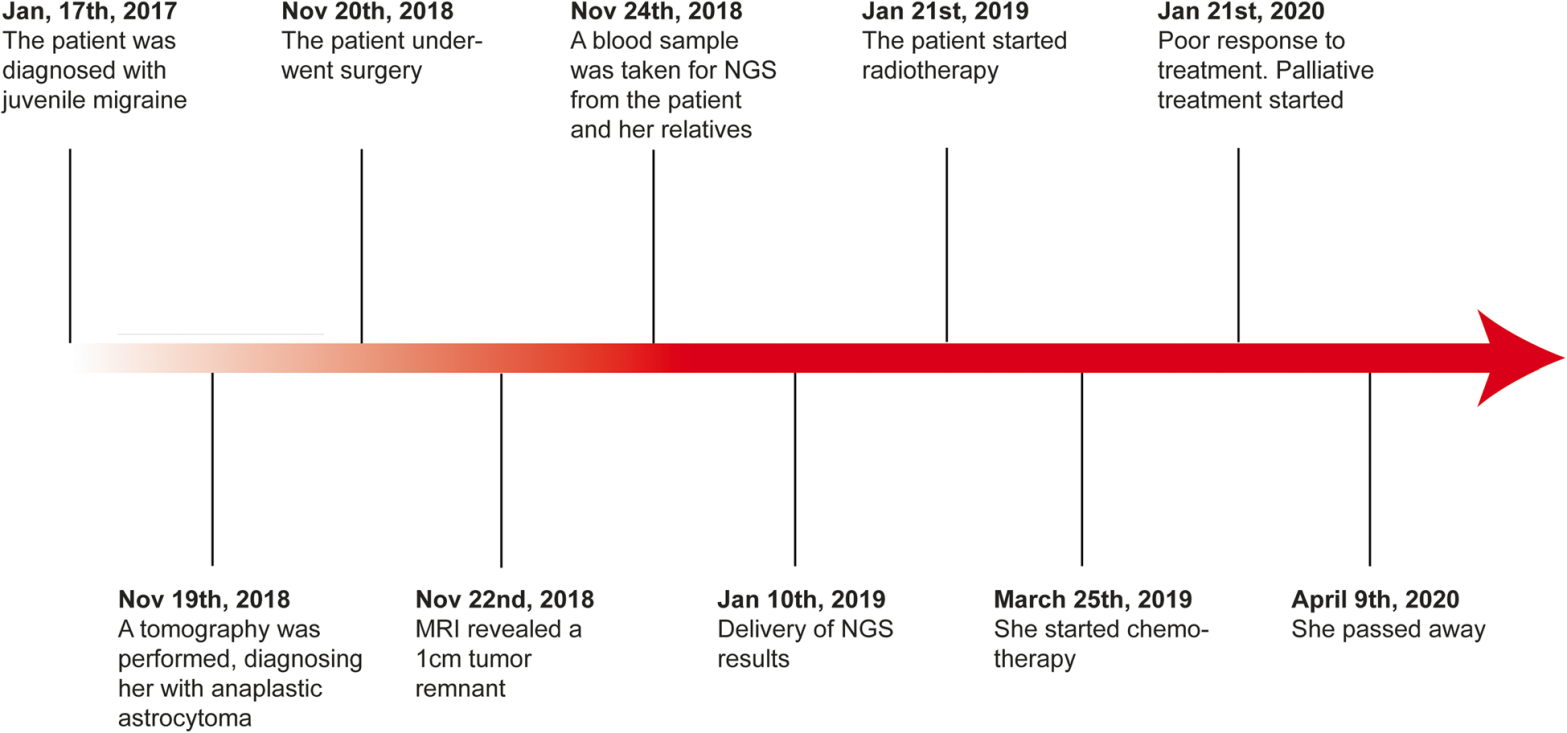
Timeline of events, interventions, and treatment

The case was analyzed in a multidisciplinary manner without benefit of reirradiation due to the risk of radionecrosis. It was classified as an unresectable tumor, and chemotherapy with irinotecan + bevacizumab was proposed. The patient and her relatives refused to continue with chemotherapy and proceeded with palliative treatment. After that, her general condition deteriorated until the April 9, 2020, when she died (Fig. 3).

Generally, high-grade AAs are associated with poor prognosis and the following clinical characteristics: long period of symptoms, postsurgical tumor remnant, and poor response to adjuvant therapy. Additionally, the age of presentation of symptoms aggravated the situation in our patient [18].

This study was approved by the Ethics Committee of Universidad San Francisco de Quito (2018-126E); informed assent was signed by the patient and her sibling and cousin (individuals III2 and III8, respectively; Fig. 1); and informed consent was signed by the patient’s parents.

Genomic DNA (gDNA) was prepared from peripheral blood using the PureLinkT Genomic DNA Kit (Invitrogen, Carlsbad, CA, USA), followed by DNA quantification using a Qubit 4 (Thermo Fisher Scientific, Waltham, MA, USA). gDNA of three samples (patient, sibling, and cousin) was enriched by using the TruSight Cancer (TSC) NGS panel (Illumina, San Diego, CA, USA), which includes approximately four thousand 80-mer probes targeting a total 255-kb region spanning > 1700 exons of 94 genes, and sequenced on the Illumina MiSeq platform. Raw sequence reads were processed and aligned against the human NCBI GRCh37/hg19 reference genome assembly using Burrows-Wheeler Aligner (BWA) software. The 80-mer probes target libraries with ~ 500 bp, enriching fragments of 350–650 bases placed in the midpoint of the probe, meaning coverage of exons and exon-flanking regions. The TSC coverage is ≥ 20 × on 95% of the target regions in the panel. The TSC gene list is detailed in Additional file 1. Sequencing workflow consisted of the tagmentation of gDNA to proceed with two cycles of cleanup, amplification, probe hybridization, and capture. All the steps can be found on the NGS panel manufacturer’s website.

The alignment and annotations were done with the BWA software. The Variant Calling Format was visualized by BaseSpace Variant Interpreter software (Illumina). When filtering the variants, we chose those reported by SIFT, PolyPhen, and ClinVar as deleterious, possibly damaging, probably damaging, pathogenic, of uncertain significance, and blanks, even if there was no coincidence between the tree sources (that is, at least one of them reported as pathogenic). We also eliminated those variants with low coverage. We proceeded in the same manner for the three individuals analyzed.

**Fig. 4:**
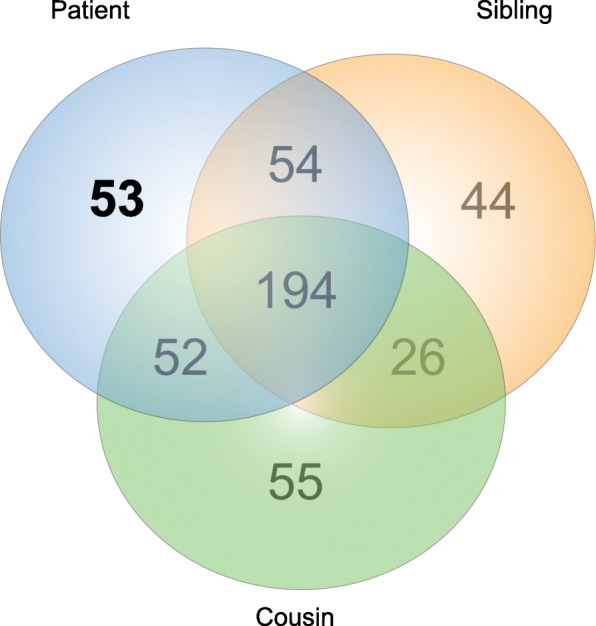
Venn diagram illustrating overlaps between probands genetic alterations found by next-generation sequencing analysis

**Fig. 5:**
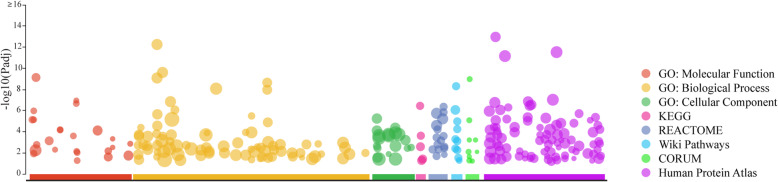
Gene set enrichment analysis using g:Profiler (https://biit.cs.ut.ee/gprofiler/). The size of the circle represents the number of genes overrepresented in certain types of molecular function or biological processes concerning Gene Ontology (molecular function, biological process, and cellular component), KEGG, REACTOME, Wiki Pathways, CORUM, and Human Protein Atlas. *P* value was adjusted (Padj) for multiple testing using the Benjamini-Hochberg method

Figure 4 shows all the genetic variants found in the three individuals analyzed. They shared 194 genetic alterations; the patient presented more variants than her sibling and her cousin (354, 319, and 328, respectively). Raw data are available in Additional file 1. Besides, the patient has 53 unique variants, which were used to perform an enrichment analysis in g:Profiler (https://biit.cs.ut.ee/gprofiler/gost; Fig. 5). These variants were related to damaged DNA binding, mismatch repair complex binding, cellular response to DNA damage stimulus, DNA recombination, and so forth (see Additional file 2). In order to identify a possibly new or existing drug developed to treat AA or any other disease that needed to target the patient’s genetic variants, we searched in the Open Target Platform (https://www.targetvalidation.org). Among the clinical trials that target alterations associated with AA, there were 96 targets, where only 4 of them targeted the patient alterations: *RET*, *EGFR*, *MET*, and *CDK4*. Although we found 13 clinical trials for 7 drugs, only 2 of the clinical researches were in children (Table 1). In the same platform, we conducted a search for drugs that targeted all the patient variants, finding 118 drugs. However, they were aimed at other diseases, not specifically AA (see Additional file 3), such as non-Hodgkin lymphoma, gastric carcinoma, clear cell sarcoma, colonic neoplasm, and asthma, among others.

**Table 1:** Drugs for anaplastic astrocytoma targeting patient genetic alterations

Target population	Drug	Drug ID	Phase	Status	Type	Mode of action	Activity	Target	Target ID
Adults	Sunitinib	CHEMBL535	2	Completed	Small molecule	Inhibitor	Antagonist	*RET*	ENSG00000165731
Adults	Vandetanib	CHEMBL24828	2	Completed	Small molecule	Inhibitor	Antagonist	*RET*	ENSG00000165731
Adults	Sunitinib	CHEMBL535	2	Completed	Small molecule	Inhibitor	Antagonist	*RET*	ENSG00000165731
Adults	Sorafenib	CHEMBL1336	1	Completed	Small molecule	Inhibitor	Antagonist	*RET*	ENSG00000165731
Adults	Sorafenib	CHEMBL1336	1	Completed	Small molecule	Inhibitor	Antagonist	*RET*	ENSG00000165731
Adults	Vandetanib	CHEMBL24828	2	Completed	Small molecule	Inhibitor	Antagonist	*EGFR*	ENSG00000146648
Adults	Erlotinib	CHEMBL553	1	Completed	Small molecule	Inhibitor	Antagonist	*EGFR*	ENSG00000146648
Adults	Erlotinib	CHEMBL553	1	Completed	Small molecule	Inhibitor	Antagonist	*EGFR*	ENSG00000146648
Adults	Erlotinib	CHEMBL553	1	Completed	Small molecule	Inhibitor	Antagonist	*EGFR*	ENSG00000146648
Adults	Cetuximab	CHEMBL1201577	1	Completed	Antibody	Inhibitor	Antagonist	*EGFR*	ENSG00000146648
Adults	Erlotinib	CHEMBL553	1	Terminated	Small molecule	Inhibitor	Antagonist	*EGFR*	ENSG00000146648
Children	Cabozantinib	CHEMBL2105717	2	Recruiting	Small molecule	Inhibitor	Antagonist	*MET*	ENSG00000105976
Children	Ribociclib	CHEMBL3545110	1	Recruiting	Small molecule	Inhibitor	Antagonist	*CDK4*	ENSG00000135446

## Discussion

In this case report, we report, for the first time, to our knowledge, panel-based NGS to characterize the genetic structure of a pediatric Ecuadorian mestizo patient with AA. As mentioned before, most of the massive population genomic studies, biobanks, cell lines, and clinical trials have not taken into account ethnically diverse populations; they have merely focused on Caucasian populations. Hence, a bias exists, generating a gap for the identification of key genes or genetic alterations present in other populations, such as mestizos [22]. This study increases knowledge in basic cancer research. Furthermore, we were able to detect a rare genetic syndrome running in the patient’s family: LFS, caused by a mutation in the *TP53* gene, which the patient inherited through her maternal ancestral line. Panel-based NGS technology allowed us to detect in her sibling and cousin the same genetic alteration, and now they are under constant surveillance.

Globally, brain tumors are the principal cause of cancer-related death in children. However, for several years, treatment remained as the same cytotoxic chemotherapy and radiation therapy, resulting in significant morbidity from both disease and treatment [6]. Additionally, survivors usually exhibited intellectual deficits and diminished quality of life [6, 23]. Currently, NGS, microarrays for epigenetic changes, and copy number arrays have broadened understanding of tumorigenesis and progression [6, 14]. High-throughput genomic platforms have also propelled novel therapies, which could provide better outcomes for children [6]. Worldwide initiatives such as the 1000 Genomes Project [24], International HapMap Project [25], Encyclopedia of DNA Elements Project (ENCODE) [26], Pan-Cancer Atlas [27], and many more have identified and compiled large numbers of genetic variants that contribute to disease risk and several lines of clinical information that allow us to access and perform population analyses. Knowledge of genetic predisposition variants and genetic alterations has a direct effect on clinical management by directing cancer care. It also enables early detection in presymptomatic relatives, timely genetic counseling, and adequate measures for cancer prevention and surveillance [28]. Nonetheless, clinical trials in children are rare and in most pediatric specialties remain unexplored. Frequently, the drugs prescribed for children are extrapolated from trials in adults, having sometimes unpredictable and tragic effects [29, 30]. Children are a diverse group, from preterm neonates to postpubertal adolescents, who have complex biological, psychological, pharmacological, and age-related characteristics different from adults [29, 31]. Thus, genome-sequencing studies have attempted to improve tumor classification and identify potential therapeutic targets [3, 4]. *In silico* analysis identified two drugs that are currently being studied in a clinical trial recruiting children.

### Deep analysis using panel-based NGS

**Fig. 6:**
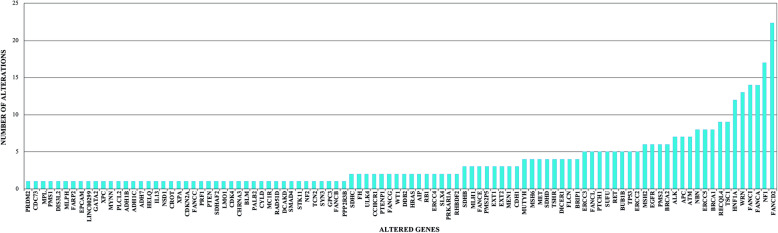
Number of genetic alterations per gene of the patient

According to ClinVar, SIFT, and PolyPhen, the patient harbored 354 genomic alterations in 100 genes. These variants were mostly implicated in DNA repair (see Additional file 2), being the top five most altered genes: *FANCD2*, *NF1*, *FANCA*, *FANCI*, and *WRN* (Fig. 6)*.* Concerning the patient’s deleterious alterations, we found rs11540652 located in *TP53* (tumor suppressor gene on chromosome 17p13; OMIM 191170), which has been classified as pathogenic, and several reports have related it to LFS (OMIM 151623) [32–36]. Germline mutations in *TP53* have been identified in 80% of patients with LFS. This gene has 11 exons; 75% of the patients have presented alterations on exons 5, 6, 7, and 8, the core DNA-binding region of the gene [37–40]. rs11540652 is located on exon 7; a change in the position c743G>A produces a missense variant from codon 248 (arginine to glutamine).

LFS is a rare disorder that increases cancer predisposition in children and young adults. The types of cancer more commonly associated are breast cancer (our patient’s grandmother and mother had it; Fig. 1), osteosarcoma, brain tumors (four members of the family), leukemia, and adrenal cortical carcinomas [37, 38, 41]. This alteration is also present in our patient’s sister and cousin (see Additional file 1). LFS has an autosomal dominant inheritance pattern, agreeing with the patient’s genealogical tree (Fig. 1). The probability of developing multiple primary tumors by the age of 30 is 50–56%, and by the age of 60, the risk increases to 90–100%. An individual with LFS has an estimated risk of 57% of developing a second cancer by the age of 30 [40, 41]. In addition to this, LFS has significant sex differences; the lifetime risk of cancer is almost 100% in women and is about 73% for men [42]. Consequently, the patient and her family present a high risk of developing cancer at any point of their lives.

Currently, clinical management of patients with LFS continues to be a challenge, given that LFS-associated genetic and epigenetic backgrounds remain mostly unknown [37, 40]. However, Villani *et al*. have shown an increase in long-term survival in patients with LFS through a comprehensive surveillance protocol that enables early tumor detection, allowing prompt tumor excision and treatment [38].

## Conclusions

NGS approaches for identifying cancer-related DNA alterations are routinely used in developed countries. However, developing countries, such as Ecuador, are far from the use of this technology in clinical practice. For instance, using NGS approaches, we found that our patient had an extensive family history of a dominant inheritance syndrome; had the family known this information sooner, it might have helped them foresee the development of AA (Fig. 1). This is only an example of how many syndromes and rare diseases are misdiagnosed, and molecular tools should be implemented in routine clinical practice for early detection and effective preemptive intervention delivery and treatment.

## Supplementary information

**Additional file 1:** TruSight Cancer Next-Generation Sequencing raw data.

**Additional file 2: Supplementary Table 2.** g:Profiler heat map.

**Additional file 3:** Nonspecific drugs for anaplastic astrocytoma.

## Data Availability

All data generated or analyzed during this study are included in this published article and its supplementary information files.

## Abbreviations

AA: Anaplastic astrocytoma
BWA: Burrows-Wheeler Aligner
gDNA: Genomic DNA
LFS: Li-Fraumeni syndrome
MRI: Magnetic resonance imaging
NGS: Next-generation sequencing
OS: Overall survival
TSC: TruSight Cancer
WHO: World Health Organization
